# Evaluation of radiation dose to pediatric models from whole body PET/CT imaging

**DOI:** 10.1002/acm2.13545

**Published:** 2022-02-02

**Authors:** Najmeh Mohammadi, Parisa Akhlaghi

**Affiliations:** ^1^ Faculty of Sciences, Physics Department Sahand University of Technology Tabriz Iran; ^2^ Faculty of Medicine, Department of Medical Physics Tabriz University of Medical Sciences Tabriz Iran

**Keywords:** dosimetry, Monte Carlo, pediatrics, PET/CT, voxel phantoms

## Abstract

Positron emission tomography (PET)/computed tomography (CT) is a well‐known modality for the diagnosis of various diseases in children and adult patients. On the other hand, younger patients are more radiosensitive than adults, so there are concerns about the level of ionizing radiation exposure in pediatric whole body PET/CT imaging. In this regard, comprehensive specific radiation dosimetry for whole body PET/CT imaging is highly desired for different ages, sizes, and shapes. Therefore, in this study, organ absorbed doses were evaluated for pediatric voxel models from 4 to 14 years old and compared with those of ICRP phantoms. Monte Carlo calculation was performed to evaluate *S*‐value, absorbed dose, and effective dose from ^18^F‐FDG radiotracers and whole body CT scan for different computational models, including 4‐ to 14‐year‐old phantoms. The results showed that the *S*‐value and, therefore, absorbed dose of ^18^F‐FDG strongly depended on the phantom anatomy. These variations were justified by the distance between source and target organs. Moreover, on average, the absorbed doses from whole body CT scans were 13.5 times lower than those from ^18^F‐FDG for all organs. According to the results, various anatomies and ages should be considered for accurate dose evaluation. These data can be used for specific risk assessment of the pediatric population in clinical nuclear imaging.

## 1 INTRODUCTION

Since its introduction, the clinical use of dual‐modality positron emission tomographic/computed tomographic (PET/CT) imaging has grown substantially. PET uses a radiopharmaceutical to provide functional data, whereas CT provides anatomical information that increases the diagnostic yield by measuring the attenuation corrections.^1^ Therefore, the main concept of combined whole body PET/CT imaging is to acquire anatomic and metabolic information in a single study;^2^ so that the incorporation of anatomic information provided by CT greatly enhances the interpretation of PET information.^3^, ^4^ Due to its advantages, the major scanner manufacturers no longer offer a PET scanner without a CT component.^5^

Because of its high sensitivity and target specificity for the pediatric population, PET/CT is well established for accurate disease detection, characterization, and treatment monitoring^6^ and provides valuable diagnostic information that may not be easily obtained using other imaging techniques for both oncologic and nononcologic indications.^7^, ^8^, ^9^ Although in the majority of cases, the medical benefit of this imaging exceeds the risk, one should be prudent when exposing children to ionizing radiation.^10^, ^11^ Consequently, it is essential to evaluate the amount of PET/CT radiation dose received in different organs and tissues within the patient's body especially for the pediatric population.

As widely accepted, children are more radiosensitive than adults due to their rapid cell division rates.^1^, ^5^, ^12^ In addition, they have the potential for a longer postirradiation life span for the emergence of induced cancer relative to the adults.^13^, ^14^, ^15^ Therefore, at the same level of radiation dose, children may experience greater stochastic risks from ionizing radiation.^16^ It was reported that lifetime attributable risks of all cancer incidence and mortality for a 10‐year‐old (male and female) are almost 5.29 and 3.16 times higher than those of an adult at age of 70 years, respectively, and these risks are even higher for younger children.^17^

In this regard, the accurate assessment of radiation dose delivered to the younger age group resulting from PET/CT is of paramount importance. Thus, some researchers evaluated the dose of this procedure for children.^18^, ^19^ According to the literature, there are considerable differences between doses reported from stylized and voxel‐based phantoms with different ages and anatomies.^16^, ^20^, ^21^ This means that the differences in the whole body properties of various individuals cause uncertainties in the dose values obtained using reference phantoms. Although reference phantoms are valuable, they have limited use in assigning organ doses for the individual patient with a body’ shape and size far from the 50th height/weight percentile. Under these conditions, phantoms of non‐50th percentile heights/weights were designed for patient dose estimates.^22^

Therefore, the assessment of organ absorbed dose and effective dose for whole body PET/CT using various pediatric computational phantoms is desired. To this end, this article will discuss the dosimetry aspect of PET/CT for reference and nonreference pediatric phantoms with different ages, including both PET, using ^18^F‐fluoro‐2‐deoxy‐D‐glucose (FDG), radiopharmaceutical, and CT components of the whole body scan to determine the effect of the anatomical discrepancies on organ absorbed doses and effective doses. Accordingly, a richer set of dosimetry results for pediatric PET (FDG)/CT studies is provided that would allow the practicing medical physicist to choose a model that more closely resembles his/her patient. The results of PET dosimetry are compared with those of ICRP pediatric reference phantoms with the same ages. Organ absorbed doses and effective doses are estimated using computational phantoms and MCNP Monte Carlo code.

## 2 MATERIALS AND METHODS

### 2.1 Computational phantoms

**FIGURE 1:**
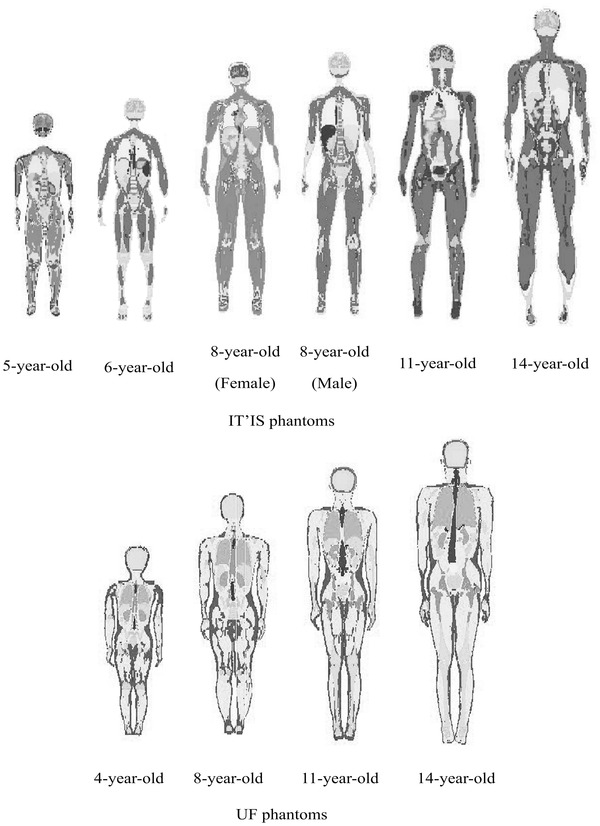
The anterior view of considered pediatric voxel phantoms in Monte Carlo simulation

Computational phantoms series B of University of Florida (UF),^23^ including 4‐, 8‐ 11‐, and 14‐year‐old male and female models, were used in this study for Monte Carlo‐based radiation dosimetry calculations. These phantoms were created from earlier head–torso phantoms of UF Series A by utilizing segmented and rescaled CT images of a healthy adult volunteer.^24^ Moreover, the virtual phantom family of Foundation for Research on Information Technologies in Society (IT'IS), including 5‐, 6‐, 8‐, 11‐, and 14‐year‐old male and female models, were considered in simulations.^25^ They were designed based on high‐resolution magnetic resonance images of healthy volunteers. As reported, UF pediatric series are representative of reference pediatric subjects but virtual phantoms of IT'IS Foundation are not in the 50th percentile of all children (worldwide). Body weights, heights, and voxel dimensions for these pediatric phantoms are summarized in Table 1. The anterior views of these computational phantoms are displayed in Figure 1.

**TABLE 1:** Characteristics of the used computational phantoms

	Age	Sex	Height (cm)	Weight (kg)	Voxel dimension (mm)	Number of tissue/organs
UF	4	Female	105.5	16.646	0.9 × 0.9 × 5.0	73
	8	Female	132	28.372	1.1 × 1.1 × 6.0	73
	11	Male	151.2	33.532	0.9 × 0.9 × 6.0	72
	14	Male	169.7	48.741	1.1 × 1.1 × 6.7	72
IT'IS	5	Female	109	17.8	5.0 × 5.0 × 5.0	66
	6	Male	117	19.3	5.0 × 5.0 × 5.0	67
	8	Female	136	30.7	5.0 × 5.0 × 5.0	75
	8	Male	139	26.0	5.0 × 5.0 × 5.0	66
	11	Female	147	35.4	5.0 × 5.0 × 5.0	75
	14	Male	169	50.4	5.0 × 5.0 × 5.0	77

### 2.2 ^18^F‐FDG radiotracers

The radiotracer investigated in this work was ^18^F‐FDG. ^18^F‐FDG is a common PET tracer. It is a glucose analog used in the identification of glucose metabolism for recognition or follow up of patients, and for investigation of myocardial and cerebral glucose metabolism.^26^ ^18^F decays by positron emission and has a half time of 109.7 min.^27^

### 2.3 Internal dosimetry calculations

The mean absorbed dose *D_T_* (in terms of mGy/MBq)delivered to the target organ or tissue (*T*) is the sum of absorbed dose arising from nuclear transformations of the radionuclide in various source organs (*S*) and is given by:

(1) $$D_{T}\;=\sum_{S}\tilde{A_{S}}\;S\;\left(T\leftarrow S\right),$$

where $\tilde{A_{S}}\;$ is the time‐integrated cumulated activity of the radiopharmaceutical in the source region and *S*(*T* ← *S*) is the *S*‐value describing the equivalent dose rate in the target organ per unit activity in the source organ.^28^ The amount of *S*(*T* ← *S*) depends on the radiation type, the energy emitted per transformation, the mass of the target organ, and the geometry of the phantoms used to represent the adult and children of various ages. The *S*‐value (in terms of mGy/MBq. h) can be calculated by:

(2) $$S\;\left(T\leftarrow S\right)=\frac{1}{M_{T}}\sum_{i}E_{i}Y_{i}\varphi_{i},$$

where *M_T_* is the mass of the target organ, *E_i_* is the energy of the *i*th radiation, *Y_i_* is the yield of *i*th radiation per nuclear transformation, and $\varphi_{i}$ is the absorbed fraction of energy of radiation type *i*, which is given by:

(3) $$\varphi_{i}=\frac{E_{d}}{E_{i}},$$

where $E_{d}$ is the energy deposited in the target tissue. $\tilde{A_{S}}$ and source organs of ^18^F‐FDG were derived from the biokinetic model reported in ICRP publication 128.^26^ These data are listed in Table 2. Brain, lungs, heart wall, liver, urinary bladder contents, and the rest of the body were considered as the source regions. It should be noted that $\tilde{A_{S}}$ value for bladder content source was considered as 0.23 for ages of 5‐ and 6‐year‐old and for other ages was set equal to 0.26. In this investigation, whole body ^18^F‐FDG PET imaging of pediatric patients with a weight‐based injected activity of 6 MBq/kg was considered.

**TABLE 2:** Biokinetic data for ^18^F‐FDG taken from ICRP publication 128

Organ (*S*)	$\tilde{A_{S}}/A_{0}$(h)
Brain	0.21
Heart wall	0.11
Lungs	0.079
Liver	0.13
Other organs and tissues	1.7
Urinary bladder contents	
Adult, 15 years, 10 years	0.26
5 years	0.23
1 year	0.16

### 2.4 CT simulation

Simulations were performed for Siemens Somatom Sensation 16 scanner (Siemens Medical Systems, Germany), which has a fan beam angle of 52° and a focal spot‐to‐axis distance of 57 cm. Scanner's characteristics and X‐ray spectra were provided by the manufacturer. The method described by Khursheed et al.^29^ was applied for simulating X‐ray source rotating around the subject. To model the CT scanner, 18‐line sources were considered around the circle (with a radius of 57 cm) and parallel to the axis of rotation. Therefore, a series of contiguous transverse slices with a thickness of 1 cm covered the scan range, and the X‐ray photons were emitted over 360°, normal to each line source. The accuracy of the simulation was previously validated by comparing measured computed tomography dose index values with those obtained by simulation.^30^, ^31^ The CT technique was used for attenuation correction and anatomic correlation of the PET findings as stated by Alessio et al.^32^ Therefore, pitch of 1, tube voltage of 120 kVp, and weight‐based tube loadings were studied for whole body PET/CT imaging of pediatric patients. Accordingly, tube loadings of 20 mAs for 4, 5, and 6 years old, 25 mAs for 8 years old, and 30 mAs for 11 and 14 years old phantoms were considered in the simulations.^1^, ^19^, ^32^, ^33^

### 2.5 Monte Carlo simulations

In this study, all the simulations were performed by MCNP‐4C Monte Carlo N‐particle code.^34^ The decay data of the investigated radionuclide were obtained from the medical internal radiation dose.^27^ The energy spectrum of positron was manually defined in MCNP input file. Radionuclide source of ^18^F was considered uniformly distributed in six source organs of brain, lungs, heart wall, liver, urinary bladder contents, and rest of the body in separated input files.

The energy deposition in the target regions was obtained in units of MeV/g per particle by +F6 tally. For numeric computations, an in‐house FORTRAN code was developed to extract the results from MCNP output files and to calculate the *S*‐values as well as the absorbed doses and effective doses using published biokinetic data and tissue weight factors. In all the simulations, 1e7 particles were transported and the relative errors of tally outputs were less than 2% in most organs.

### 2.6 Effective dose calculation

Equivalent dose is a dose quantity representing the impact of radiation type on organs and tissues, and is defined as the absorbed radiation dose in an organ/tissue corrected by a radiation weighting factor. The equivalent dose of *H_T_* is calculated as *H_T_*_ _= *W_R_* ×*D_T_*; where *W_R_* is the radiation weighting factor for radiation *R*. It should be noted that *W_R_* of positron radiation is equal to 1. Thus, *H_T_* is equal to the absorbed dose for this radiation.

To estimate the effect of equivalent doses on all organs of the human body, the concept of the effective dose was introduced by ICRP publication 103.^35^ The effective dose is defined by weighted sum of tissue equivalent doses as $E=\sum_{T}W_{T}H_{T}$; where $W_{T}$ is the tissue weighting factors for target organ *T*, and $H_{T}$ is the equivalent dose in the target organ *T*.

## 3 RESULTS

### 3.1 CT absorbed dose

Absorbed doses of 30 organs (in mGy/mAs) for UF and IT'IS phantoms at a tube voltage of 120 kVp are listed in Table 3. It should be mentioned that eye lenses, breast, thyroid, salivary gland, adrenals, and pituitary gland for some phantoms were not defined in their models. So, absorbed doses for these organs were not reported. Based on the table, the organ absorbed doses depended on the ages and anatomies of phantoms. For instance, colon and lung doses of all UF phantoms were higher than those of IT'IS at the same age with the maximum difference of 6.2% (for 8 years) and 12.4% (for 14 years), respectively. On the other hand, the brain dose of UF 8‐year‐old phantom was 5.4% greater and 0.6% lower than that of IT'IS 8‐year‐old female and male phantoms, separately. While this value for UF 11‐year‐old was 7.8% lower than that of IT'IS 11‐year‐old. In addition, the brain dose difference between 14‐year‐old phantom models was 2.6%.

**TABLE 3:** Organ absorbed dose in whole body CT imaging (mGy/mAs) for different phantoms at 120 kVp

	UF				IT'IS					
Age (year)	4	8	11	14	5	6	8(F^*^)	8(M^**^)	11	14
Breast	3.25E‐2	3.26E‐2	–	–	–	–	–	–	–	–
Colon	4.01E‐2	3.86E‐2	3.63E‐2	3.47E‐2	3.81E‐2	3.68E‐2	3.66E‐2	3.62E‐2	3.53E‐2	3.37E‐2
Lungs	4.06E‐2	3.85E‐2	3.77E‐2	3.80E‐2	3.88E‐2	3.66E‐2	3.57E‐2	3.79E‐2	3.47E‐2	3.33E‐2
Stomach	3.75E‐2	3.82E‐2	3.53E‐2	3.30E‐2	3.67E‐2	3.57E‐2	3.55E‐2	3.69E‐2	3.47E‐2	3.40E‐2
Urinary bladder	4.19E‐2	3.94E‐2	3.67E‐2	3.53E‐2	4.16E‐2	3.74E‐2	3.41E‐2	3.78E‐2	3.37E‐2	3.28E‐2
Liver	3.83E‐2	3.77E‐2	3.57E‐2	3.37E‐2	3.80E‐2	3.61E‐2	3.63E‐2	3.72E‐2	3.53E‐2	3.50E‐2
Esophagus	4.38E‐2	3.93E‐2	3.90E‐2	4.00E‐2	6.95E‐2	4.48E‐2	3.38E‐2	4.52E‐2	3.63E‐2	3.87E‐2
Thyroid	9.15E‐2	9.16E‐2	5.77E‐2	6.17E‐2	–	–	–	–	8.60E‐2	9.37E‐2
Gonads	3.87E‐2	3.64E‐2	5.27E‐2	4.87E‐2	3.74E‐2	7.05E‐2	3.14E‐2	7.72E‐2	2.81E‐2	6.67E‐2
Skin	5.90E‐2	5.84E‐2	5.90E‐2	5.77E‐2	5.55E‐2	5.70E‐2	5.96E‐2	6.12E‐2	6.03E‐2	5.93E‐2
Brain	6.85E‐2	6.60E‐2	6.00E‐2	6.17E‐2	6.35E‐2	6.55E‐2	6.24E‐2	6.64E‐2	6.47E‐2	6.33E‐2
Kidney	3.92E‐2	3.88E‐2	3.57E‐2	3.37E‐2	3.73E‐2	3.61E‐2	3.60E‐2	3.61E‐2	3.47E‐2	3.40E‐2
Salivary glands	7.15E‐2	7.40E‐2	6.97E‐2	6.93E‐2	–	–	–	–	–	–
Adipose	5.20E‐2	5.04E‐2	5.10E‐2	4.77E‐2	4.87E‐2	4.67E‐2	4.20E‐2	4.32E‐2	4.77E‐2	4.10E‐2
Adrenals	3.35E‐2	3.31E‐2	3.07E‐2	2.97E‐2	–	3.24E‐2	3.07E‐2	–	2.99E‐2	2.75E‐2
ET region	9.30E‐2	9.04E‐2	8.87E‐2	8.57E‐2	6.85E‐2	5.35E‐2	4.92E‐2	5.84E‐2	5.93E‐2	6.73E‐2
Gall bladder	3.52E‐2	3.69E‐2	3.37E‐2	3.08E‐2	3.64E‐2	3.56E‐2	3.47E‐2	3.51E‐2	3.43E‐2	3.37E‐2
Heart wall	3.99E‐2	3.78E‐2	3.70E‐2	3.73E‐2	3.82E‐2	3.72E‐2	3.58E‐2	3.73E‐2	3.47E‐2	3.30E‐2
Muscle	5.90E‐2	5.84E‐2	5.67E‐2	5.53E‐2	6.05E‐2	5.75E‐2	6.12E‐2	6.20E‐2	5.77E‐2	5.93E‐2
Pancreas	3.63E‐2	3.79E‐2	3.33E‐2	3.09E‐2	3.61E‐2	3.43E‐2	3.43E‐2	3.39E‐2	3.29E‐2	3.24E‐2
SI wall	3.98E‐2	3.83E‐2	3.57E‐2	3.47E‐2	3.80E‐2	3.55E‐2	3.76E‐2	3.56E‐2	3.37E‐2	3.47E‐2
Spleen	3.89E‐2	3.88E‐2	3.70E‐2	3.43E‐2	3.86E‐2	3.70E‐2	3.70E‐2	3.89E‐2	3.57E‐2	3.63E‐2
Thymus	4.10E‐2	3.53E‐2	3.67E‐2	3.57E‐2	3.68E‐2	3.64E‐2	3.40E‐2	3.61E‐2	3.30E‐2	3.40E‐2
Uterus/prostate	3.75E‐2	3.50E‐2	3.63E‐2	3.57E‐2	3.42E‐2	3.68E‐2	3.13E‐2	4.20E‐2	2.69E‐2	3.09E‐2
Eye lenses	7.30E‐2	7.36E‐2	7.03E‐2	7.07E‐2	6.40E‐2	6.55E‐2	6.64E‐2	7.40E‐2	7.40E‐2	–
Pituitary gland	5.60E‐2	5.56E‐2	5.07E‐2	5.07E‐2	4.16E‐2	4.43E‐2	3.96E‐2	–	4.87E‐2	4.90E‐2
Spinal cord	4.34E‐2	4.16E‐2	4.27E‐2	3.87E‐2	4.03E‐2	3.64E‐2	4.32E‐2	3.51E‐2	3.43E‐2	4.17E‐2
Red bone marrow	6.05E‐2	5.56E‐2	5.37E‐2	5.17E‐2	5.90E‐2	6.50E‐2	5.72E‐2	5.48E‐2	5.63E‐2	4.37E‐2

^*^Female.

^**^Male.

### 3.2 *S*‐values

**FIGURE 2:**
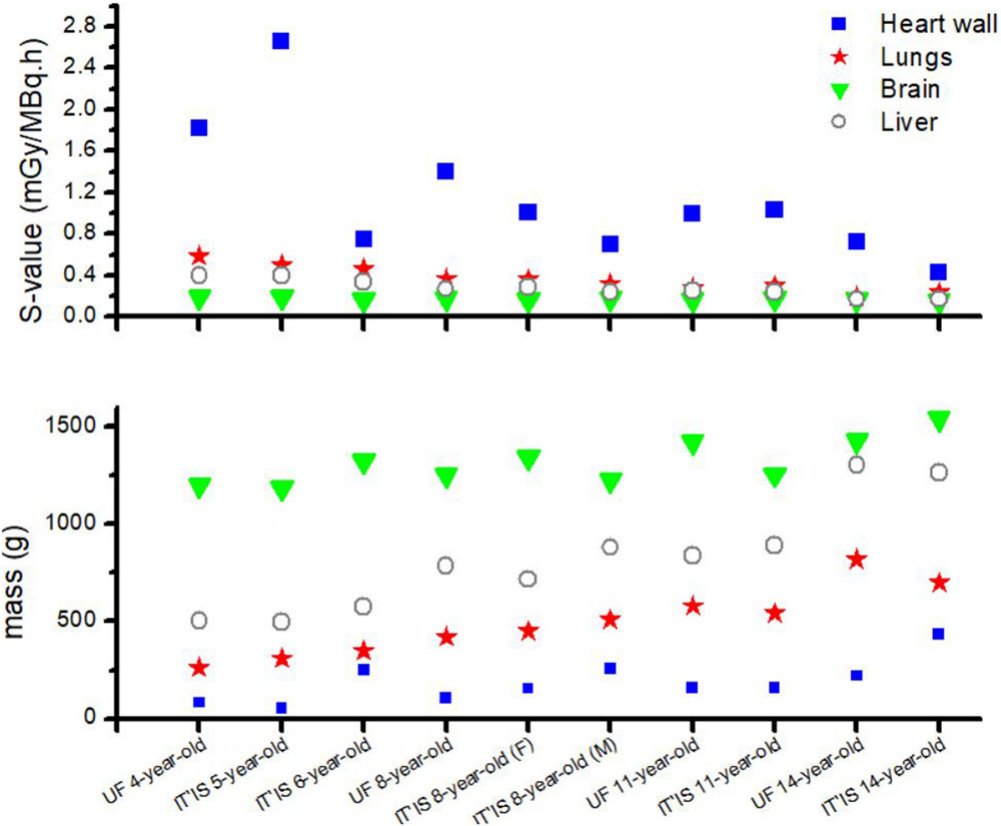
Self‐absorbed dose *S*‐value for UF and IT'IS phantoms

*S*‐values of ^18^F‐FDG for the source organs, including brain, liver, bladder content, lungs, heart wall, and rest of the body, were calculated in the considered computational phantoms in terms of mGy/MBq.h. The *S*‐values of critical target organs for considered phantoms are given in Tables 4, 5, 6, 7, 8, 9 for UF and IT'IS phantoms. Since positrons deposited their energies at a short distance from their creation locations; when the source and target organs were the same, its absorbed dose (self‐absorbed dose) *S*‐value was higher than the absorbed dose of other target organs. Moreover, by increasing the age and organ dimension, the self‐absorbed dose *S*‐value decreased. For example, the self‐absorbed dose *S*‐value of the brain ranged from 1.86E‐1 to 1.61E‐1 mGy/MBq.h for UF pediatric phantoms. Whereas, this value for IT'IS phantoms was from 1.86E‐1 to 1.49E‐1 mGy/MBq.h. A similar behavior was found for the other source organs. The self‐absorbed dose *S*‐values of the source organs and their masses for different ages of phantoms are displayed in Figure 2. According to the results, the self‐absorbed dose *S*‐values decreased when the source organs mass increased. As known, absorbed dose is energy deposition per unit mass. So, younger patients with smaller organs received higher doses. So that, heart wall of IT'IS 5‐year‐old had the lowest mass and the highest self‐absorbed dose *S*‐value. Also, the self‐absorbed dose *S*‐value of heart for UF 8‐year‐old was higher than those for IT'IS 8‐year‐old male and female about 39% and 98%, respectively. This amount was 9.95E‐1 and 1.03 mGy/MBq.h for 11‐year‐old UF model and IT'IS phantom, respectively. While for 14‐year‐old, the heart *S*‐value of IT'IS phantom was 70% lower than that of UF phantom.

**TABLE 4:** The *S*‐value of lungs as source for different target organs

	UF				IT'IS					
Age (year)	4	8	11	14	5	6	8(F^*^)	8(M^**^)	11	14
Skin	4.20E‐3	2.71E‐3	2.14E‐3	1.70E‐3	5.09E‐3	3.53E‐3	2.64E‐3	2.93E‐3	2.69E‐3	1.75E‐3
Stomach	2.16E‐2	1.41E‐2	1.45E‐2	8.07E‐3	2.29E‐2	1.60E‐2	1.11E‐2	1.22E‐2	7.88E‐3	5.23E‐3
Brain	1.94E‐3	1.03E‐3	9.44E‐4	6.08E‐4	1.23E‐3	1.24E‐3	9.48E‐4	7.48E‐4	6.93E‐4	5.10E‐4
Gall bladder	8.00E‐3	5.93E‐3	8.09E‐3	4.26E‐3	7.11E‐3	5.68E‐3	6.40E‐3	4.55E‐3	3.33E‐3	2.41E‐3
Thyroid	2.19E‐2	1.16E‐2	1.75E‐2	1.19E‐2	–	–	–	–	1.12E‐2	6.11E‐3
Heart	4.59E‐2	3.21E‐2	3.31E‐2	2.36E‐2	5.35E‐2	4.23E‐2	3.40E‐2	3.30E‐2	2.97E‐2	2.05E‐2
Liver	1.87E‐2	1.50E‐2	1.41E‐2	6.56E‐3	2.03E‐2	1.49E‐2	1.26E‐2	1.30E‐2	1.14E‐2	7.04E‐3
Bladder	8.75E‐4	3.46E‐4	2.24E‐4	2.66E‐4	6.03E‐4	4.82E‐4	3.69E‐4	2.10E‐4	1.49E‐4	1.61E‐4
Small intestine	3.73E‐3	2.31E‐3	2.14E‐3	1.15E‐3	4.31E‐3	6.71E‐3	2.45E‐3	3.74E‐3	5.73E‐4	1.04E‐3
Pancreas	9.64E‐3	7.44E‐3	7.89E‐3	4.75E‐3	1.05E‐2	8.22E‐3	6.80E‐3	4.70E‐3	3.55E‐3	3.21E‐3
Thymus	3.91E‐2	2.90E‐2	3.17E‐2	2.21E‐2	4.31E‐2	4.32E‐2	3.14E‐2	3.66E‐2	3.30E‐2	1.74E‐2
Kidney	8.63E‐3	5.63E‐3	7.33E‐3	6.81E‐3	7.64E‐3	5.87E‐3	6.38E‐3	4.66E‐3	3.38E‐3	3.89E‐3
Colon	2.63E‐3	1.67E‐3	1.90E‐3	1.80E‐3	4.60E‐3	2.39E‐3	2.08E‐3	1.23E‐3	7.91E‐4	8.06E‐4
Lungs	5.88E‐1	3.74E‐1	2.77E‐1	1.95E‐1	5.03E‐1	4.56E‐1	3.58E‐1	3.19E‐1	3.03E‐1	2.37E‐1
Adrenal	1.69E‐2	1.06E‐2	1.32E‐2	1.78E‐2	–	1.15E‐2	1.12E‐2	–	6.46E‐3	8.43E‐3
Red bone marrow	8.65E‐3	6.16E‐3	5.17E‐3	3.25E‐3	1.02E‐3	1.51E‐3	1.36E‐3	1.17E‐3	3.56E‐4	3.28E‐4
Extrathoracic airways	1.20E‐2	6.78E‐3	2.53E‐2	4.58E‐3	9.16E‐3	2.36E‐2	1.59E‐2	1.25E‐2	1.28E‐2	8.95E‐3

^*^Female.

^**^Male.

**TABLE 5:** The *S*‐value of brain as source for different target organs

	UF				IT'IS					
Age (year)	4	8	11	14	5	6	8(F^*^)	8(M^**^)	11	14
Skin	3.16E‐3	2.34E‐3	1.98E‐3	1.49E‐3	2.16E‐3	2.08E‐3	1.66E‐3	1.58E‐3	1.72E‐3	7.44E‐4
Stomach	6.07E‐4	3.09E‐4	3.11E‐4	1.57E‐4	4.42E‐4	4.27E‐4	2.94E‐4	1.77E‐4	1.69E‐4	8.90E‐5
Brain	1.86E‐1	1.81E‐1	1.62E‐1	1.61E‐1	1.86E‐1	1.69E‐1	1.68E‐1	1.77E‐1	1.75E‐1	1.49E‐1
Gall bladder	4.39E‐4	2.14E‐4	2.86E‐4	1.22E‐4	3.23E‐4	2.17E‐4	1.45E‐4	1.68E‐4	9.02E‐5	5.35E‐5
Thyroid	6.31E‐3	3.75E‐3	2.80E‐3	2.28E‐3	–	–	–	–	2.74E‐3	2.20E‐3
Heart	1.49E‐3	7.37E‐4	7.13E‐4	4.59E‐4	8.26E‐4	9.11E‐4	6.67E‐4	4.99E‐4	4.47E‐4	2.94E‐4
Liver	7.09E‐4	3.43E‐4	3.57E‐4	1.52E‐4	4.50E‐4	4.22E‐4	3.08E‐4	2.31E‐4	2.07E‐4	1.27E‐4
Bladder	6.82E‐5	1.87E‐5	1.37E‐5	1.02E‐5	3.33E‐5	2.83E‐5	2.07E‐5	1.79E‐5	8.26E‐6	7.88E‐6
Small intestine	2.25E‐4	1.09E‐4	8.76E‐5	3.67E‐5	1.69E‐4	2.57E‐4	1.05E‐4	1.01E‐4	2.66E‐5	2.8E‐5
Pancreas	4.28E‐4	2.21E‐4	2.20E‐4	1.21E‐4	2.88E‐4	2.90E‐4	1.86E‐4	1.37E‐4	8.78E‐5	9.08E‐5
Thymus	3.19E‐3	1.17E‐3	1.57E‐3	1.12E‐3	1.34E‐3	1.68E‐3	1.46E‐3	9.23E‐4	7.51E‐4	6.62E‐4
Kidney	4.52E‐4	2.09E‐4	2.35E‐4	1.62E‐4	2.70E‐4	2.30E‐4	1.80E‐4	1.31E‐4	9.74E‐5	9.17E‐5
Colon	1.81E‐4	8.63E‐5	8.15E‐5	5.95E‐5	1.67E‐4	1.21E‐4	8.75E‐5	4.14E‐5	3.23E‐5	2.21E‐5
Lungs	1.93E‐3	1.03E‐3	9.41E‐4	6.05E‐4	1.23E‐3	1.26E‐3	9.52E‐4	7.25E‐4	7.00E‐4	5.03E‐4
Adrenal	6.02E‐4	2.81E‐4	2.96E‐4	2.47E‐4	–	3.12E‐4	3.18E‐4	–	1.43E‐4	6.65E‐5
Red bone marrow	8.67E‐3	6.83E‐3	6.01E‐3	4.74E‐3	1.12E‐4	1.21E‐2	2.05E‐3	5.51E‐3	7.12E‐3	4.88E‐5
Extrathoracic airways	1.11E‐2	8.19E‐3	2.15E‐3	6.10E‐3	1.01E‐2	7.21E‐3	4.75E‐3	6.36E‐3	5.66E‐3	2.86E‐3

^*^Female.

^**^Male.

**TABLE 6:** The *S*‐value of urinary bladder content as source for different target organs

	UF				IT'IS					
Age (year)	4	8	11	14	5	6	8(F^*^)	8(M^**^)	11	14
Skin	4.03E‐3	2.38E‐3	1.99E‐3	1.5E‐3	4.11E‐3	3.49E‐3	2.4E‐3	2.59E‐3	1.89E‐3	1.6E‐3
Stomach	3.74E‐3	1.55E‐3	6.79E‐4	1.18E‐3	1.83E‐3	1.79E‐3	1.43E‐3	1.52E‐3	9.81E‐4	1.13E‐3
Brain	6.96E‐5	2.13E‐5	1.22E‐5	9.40E‐6	3.46E‐5	3.23E‐5	1.89E‐5	1.17E‐5	7.63E‐6	5.72E‐6
Gall bladder	4.72E‐3	1.61E‐3	7.75E‐4	1.27E‐3	3.75E‐3	2.92E‐3	1.84E‐3	1.57E‐3	1.62E‐3	1.91E‐3
Thyroid	2.74E‐4	6.42E‐5	6.70E‐5	6.33E‐5	–	–	–	–	3.09E‐5	2.53E‐5
Heart	1.04E‐3	4.83E‐4	2.45E‐4	2.38E‐4	7.66E‐4	5.76E‐4	4.85E‐4	2.56E‐4	1.97E‐4	2.15E‐4
Liver	2.62E‐3	1.04E‐3	5.67E‐4	9.47E‐4	1.88E‐3	1.54E‐3	1.26E‐3	8.52E‐4	6.42E‐4	8.23E‐4
Bladder	5.00E‐1	8.29E‐1	4.42E‐1	1.30E‐1	5.98E0	4.97E0	1.24E0	3.11E0	4.21E0	9.51E‐1
Small intestine	1.00E‐2	6.79E‐3	6.05E‐3	1.45E‐2	4.60E‐2	2.94E‐3	1.12E‐2	3.01E‐3	4.11E‐2	1.36E‐2
Pancreas	4.43E‐3	1.46E‐3	9.97E‐4	1.67E‐3	2.76E‐3	2.80E‐3	2.26E‐3	2.03E‐3	1.50E‐3	1.50E‐3
Thymus	4.75E‐4	2.59E‐4	9.14E‐5	9.13E‐5	3.29E‐4	2.83E‐4	2.05E‐4	1.37E‐4	1.04E‐4	6.86E‐5
Kidney	4.14E‐3	1.73E‐3	9.75E‐4	1.16E‐3	3.47E‐3	3.25E‐3	2.14E‐3	1.68E‐3	1.41E‐3	1.14E‐3
Colon	5.00E‐2	3.68E‐2	3.21E‐2	9.19E‐3	3.09E‐2	5.30E‐2	2.32E‐2	5.28E‐2	3.03E‐2	2.27E‐2
Lungs	8.86E‐4	3.53E‐4	2.19E‐4	2.61E‐4	6.08E‐4	4.75E‐4	3.74E‐4	2.29E‐4	1.66E‐4	1.59E‐4
Adrenal	2.84E‐3	1.08E‐3	7.14E‐4	6.53E‐4	–	1.80E‐3	1.15E‐3	–	6.22E‐4	5.79E‐4
Red bone marrow	1.00E‐2	5.93E‐3	5.45E‐3	4.34E‐3	1.04E‐2	4.49E‐4	4.21E‐3	5.62E‐3	3.47E‐3	1.53E‐3
Extrathoracic airways	2.09E‐4	6.68E‐5	9.35E‐5	3.72E‐5	7.08E‐5	2.02E‐4	1.10E‐4	8.46E‐5	4.81E‐5	1.90E‐5

^*^Female.

^**^Male.

**TABLE 7:** The *S*‐value of heart wall as source for different target organs

	UF				IT'IS					
Age (year)	4	8	11	14	5	6	8(F^*^)	8(M^**^)	11	**14**
Skin	3.71E‐3	2.46E‐3	1.93E‐3	1.49E‐3	4.89E‐3	3.01E‐3	2.39E‐3	2.49E‐3	2.35E‐3	1.56E‐3
Stomach	2.48E‐2	2.15E‐2	1.74E‐2	7.47E‐3	3.86E‐2	2.23E‐2	2.40E‐2	1.44E‐2	1.54E‐2	1.16E‐2
Brain	1.49E‐3	7.11E‐4	7.08E‐4	4.53E‐4	7.97E‐4	9.14E‐4	6.78E‐4	4.88E‐4	4.40E‐4	2.78E‐4
Gall bladder	1.00E‐2	5.33E‐3	8.56E‐3	4.23E‐3	9.47E‐3	7.89E‐3	8.11E‐3	5.86E‐3	4.34E‐3	3.33E‐3
Thyroid	1.84E‐2	7.15E‐3	1.26E‐2	7.11E‐3	–	–	–	–	5.17E‐3	3.04E‐3
Heart	1.82E0	1.40E0	9.95E‐1	7.21E‐1	2.66E0	7.54E‐1	1.01E0	7.05E‐1	1.03E0	4.24E‐1
Liver	2.47E‐2	1.54E‐2	1.86E‐2	7.87E‐3	2.49E‐2	2.07E‐2	2.00E‐2	1.80E‐2	1.71E‐2	9.97E‐3
Bladder	1.00E‐3	5.02E‐4	2.39E‐4	2.39E‐4	8.06E‐4	5.73E‐4	4.94E‐4	2.53E‐4	1.96E‐4	2.09E‐4
Small intestine	4.76E‐3	3.50E‐3	2.52E‐3	1.15E‐3	6.74E‐3	9.84E‐3	3.76E‐3	5.58E‐3	8.28E‐4	1.65E‐3
Pancreas	1.48E‐2	1.17E‐2	9.44E‐3	4.33E‐3	2.11E‐2	1.21E‐2	1.31E‐2	7.35E‐3	6.24E‐3	5.36E‐3
Thymus	9.69E‐2	1.02E‐1	4.55E‐2	3.84E‐2	1.32E‐1	9.54E‐2	1.02E‐1	9.41E‐2	7.30E‐2	4.39E‐2
Kidney	8.53E‐3	5.96E‐3	5.89E‐3	4.11E‐3	8.81E‐3	6.41E‐3	8.06E‐3	5.11E‐3	4.89E‐3	4.47E‐3
Colon	3.20E‐3	2.47E‐3	2.22E‐3	1.74E‐3	7.41E‐3	3.21E‐3	2.99E‐3	1.61E‐3	1.14E‐3	1.19E‐3
Lungs	4.59E‐2	3.21E‐2	3.3E‐2	2.36E‐2	5.36E‐2	4.22E‐2	3.40E‐2	3.31E‐2	2.97E‐2	2.04E‐2
Adrenal	1.95E‐2	1.40E‐2	1.21E‐2	9.71E‐3	–	1.47E‐2	1.81E‐2	–	1.00E‐2	1.06E‐2
Red bone marrow	5.83E‐3	4.33E‐3	3.26E‐3	2.21E‐3	8.82E‐4	1.33E‐3	9.78E‐4	9.26E‐4	2.55E‐4	2.18E‐4
Extrathoracic airways	9.92E‐3	4.38E‐3	2.66E‐2	3.29E‐3	5.99E‐3	2.13E‐2	1.25E‐2	1.16E‐2	7.41E‐3	5.33E‐3

^*^Female.

^**^Male.

**TABLE 8:** The *S*‐value of liver as source for different target organs

	UF				IT'IS					
Age (year)	4	8	11	14	5	6	8(F^*^)	8(M^**^)	11	14
Skin	4.37E‐3	2.83E‐3	2.26E‐3	1.57E‐3	4.99E‐3	3.35E‐3	2.53E‐3	2.65E‐3	2.02E‐3	1.67E‐3
Stomach	2.53E‐2	2.60E‐2	2.48E‐2	1.35E‐2	2.89E‐2	2.73E‐2	2.65E‐2	1.96E‐2	1.64E‐2	1.63E‐2
Brain	7.03E‐4	3.39E‐4	3.52E‐4	1.47E‐4	4.52E‐4	4.22E‐4	3.01E‐4	2.32E‐4	2.07E‐4	1.22E‐4
Gall bladder	1.06E‐1	9.43E‐2	9.76E‐2	6.84E‐2	9.66E‐2	8.71E‐2	9.07E‐2	6.97E‐2	5.21E‐2	4.52E‐2
Thyroid	4.46E‐3	2.33E‐3	3.03E‐3	1.23E‐3	–	–	–	–	1.63E‐3	1.01E‐3
Heart	2.48E‐2	1.55E‐2	1.87E‐2	7.87E‐3	2.49E‐2	2.08E‐2	2.00E‐2	1.80E‐2	1.71E‐2	9.92E‐3
Liver	3.95E‐1	2.67E‐1	2.48E‐1	1.69E‐1	4.04E‐1	3.43E‐1	2.87E‐1	2.40E‐1	2.38E‐1	1.72E‐1
Bladder	2.57E‐3	1.04E‐3	5.84E‐4	9.66E‐4	1.85E‐3	1.52E‐3	1.27E‐3	8.28E‐4	6.27E‐4	8.64E‐4
Small intestine	1.18E‐2	8.17E‐3	7.81E‐3	6.70E‐3	1.14E‐2	2.58E‐2	1.12E‐2	1.49E‐2	2.93E‐3	5.24E‐3
Pancreas	4.30E‐2	2.99E‐2	2.46E‐2	1.15E‐2	5.95E‐2	4.44E‐2	3.55E‐2	3.22E‐2	2.41E‐2	2.04E‐2
Thymus	8.07E‐3	5.62E‐3	4.59E‐3	2.01E‐3	9.01E‐3	7.63E‐3	5.51E‐3	5.25E‐3	6.2E‐3	3.31E‐3
Kidney	3.30E‐2	2.09E‐2	1.91E‐2	1.08E‐2	3.78E‐2	3.02E‐2	2.58E‐2	2.46E‐2	2.23E‐2	1.95E‐2
Colon	1.14E‐2	5.82E‐3	9.15E‐3	1.22E‐2	1.32E‐2	1.17E‐2	1.12E‐2	7.77E‐3	5.54E‐3	5.46E‐3
Lungs	1.87E‐2	1.51E‐2	1.41E‐2	6.57E‐3	2.03E‐2	1.51E‐2	1.26E‐2	1.30E‐2	1.14E‐2	6.99E‐3
Adrenal	4.86E‐2	2.93E‐2	3.41E‐2	1.22E‐2	–	6.43E‐2	4.50E‐2	–	2.93E‐2	2.91E‐2
Red bone marrow	4.83E‐3	3.47E‐3	2.89E‐3	1.36E‐3	9.46E‐4	5.27E‐4	7.28E‐4	7.63E‐4	1.79E‐4	2.01E‐4
Extrathoracic airways	2.97E‐3	1.61E‐3	3.96E‐3	7.26E‐4	2.16E‐3	3.48E‐3	2.25E‐3	1.79E‐3	1.81E‐3	1.13E‐3

^*^Female.

^**^Male.

**TABLE 9:** The *S*‐value of rest of body as source for different target organs

	UF				IT'IS					
Age (year)	4	8	11	14	5	6	8(F^*^)	8(M^**^)	11	14
Skin	6.82E‐3	5.12E‐3	4.24E‐3	3.37E‐3	4.90E‐3	4.44E‐3	8.3.0E‐3	3.26E‐3	7.32E‐3	2.13E‐3
Stomach	1.77E‐2	1.79E‐2	1.73E‐2	1.23E‐2	2.30E‐2	2.12E‐2	1.09E‐2	1.42E‐2	9.29E‐3	8.36E‐3
Brain	3.53E‐3	2.3E‐3	2.00E‐3	1.87E‐3	3.62E‐3	1.28E‐3	2.03E‐3	3.52E‐3	1.78E‐3	1.20E‐3
Gall bladder	1.66E‐2	1.67E‐2	1.34E‐2	1.08E‐2	8.65E‐3	1.82E‐2	1.01E‐2	1.37E‐2	9.50E‐3	8.11E‐3
Thyroid	2.01E‐2	1.86E‐2	1.49E‐2	1.22E‐2	–	–	–	–	1.03E‐2	6.86E‐3
Heart	6.75E‐3	3.56E‐3	3.20E‐3	2.38E‐3	6.57E‐3	5.85E‐3	3.89E‐3	4.01E‐3	4.06E‐3	2.67E‐3
Liver	6.34E‐3	3.47E‐3	3.71E‐3	2.57E‐3	6.14E‐3	6.46E‐3	3.97E‐3	4.07E‐3	3.35E‐3	3.02E‐3
Bladder	1.97E‐2	2.12E‐2	1.69E‐2	1.20E‐2	1.12E‐2	1.06E‐2	6.10E‐3	7.33E‐3	5.88E‐3	4.43E‐3
Small intestine	2.08E‐2	2.10E‐2	1.70E‐2	1.34E‐2	2.33E‐2	2.32E‐2	1.19E‐2	1.51E‐2	1.02E‐2	8.97E‐3
Pancreas	2.09E‐2	2.16E‐2	1.89E‐2	1.45E‐2	2.43E‐2	2.33E‐2	1.20E‐2	1.57E‐2	1.01E‐2	9.44E‐3
Thymus	1.92E‐2	1.92E‐2	1.61E‐2	1.32E‐2	2.07E‐2	1.95E‐2	1.01E‐2	1.33E‐2	8.87E‐3	7.51E‐3
Kidney	2.09E‐2	2.20E‐2	1.77E‐2	1.43E‐2	2.42E‐2	2.34E‐2	1.17E‐2	1.62E‐2	8.78E‐3	8.93E‐3
Colon	1.87E‐2	1.87E‐2	1.59E‐2	1.32E‐2	2.33E‐2	2.16E‐2	1.17E‐2	1.49E‐2	1.04E‐2	8.89E‐3
Lungs	7.28E‐3	3.98E‐3	3.67E‐3	2.86E‐3	6.59E‐3	7.25E‐3	4.32E‐3	4.89E‐3	3.82E‐3	3.14E‐3
Adrenal	2.13E‐2	2.20E‐2	1.77E‐2	1.30E‐2	–	2.36E‐2	1.11E‐2	–	9.86E‐3	8.9E‐3
Red bone marrow	1.58E‐2	1.62E‐2	1.28E‐2	1.05E‐2	2.15E‐2	1.83E‐2	1.00E‐2	1.49E‐2	8.59E‐3	7.39E‐3
Extrathoracic airways	1.96E‐2	1.86E‐2	1.59E‐2	1.21E‐2	1.95E‐2	2.08E‐2	1.06E‐2	1.54E‐2	9.94E‐3	7.81E‐3

^*^Female.

^**^Male.

It can be seen that the cross‐absorbed dose *S*‐value (when the source and target organs are not the same) depended on the distance between source and target organ. According to the results, the cross‐absorbed dose *S*‐value had a complicated correlation with age and source organs; because the amount of energy deposition from positrons and photons in the target organs is more affected by source/target distance. As reported in some studies, this behavior could be justified considering the location of the source organ compared to the target organs.^16^

Given Table 5, for instance, by increasing the distance between the brain (as the source) and target organs, such as small intestine, kidneys, and colon, the cross‐absorbed dose *S*‐value decreased. Mainly, the cross‐absorbed dose *S*‐value decreased when the phantom age increased. A similar tendency was observed for other sources and target organs.

### 3.3 ^18^F‐FDG absorbed dose

The absorbed dose of target organs from ^18^F‐FDG was calculated using Equation (1). The absorbed doses per unit administered activity to critical organs for all phantoms are displayed in Table 10.

**TABLE 10:** Absorbed dose of ^18^F‐FDG (mGy/MBq.h) for UF and IT'IS phantoms

	UF				IT'IS					
Age (year)	4	8	11	14	5	6	8(F^*^)	8(M^**^)	11	14
Gonads	6.36E‐2	5.45E‐2	3.16E‐2	2.40E‐2	8.46E‐2	1.61E‐2	3.52E‐2	1.56E‐2	8.48E‐2	4.35E‐2
Skin	1.45E‐2	1.07E‐2	8.82E‐3	6.93E‐3	1.40E‐2	7.4E‐3	1.59E‐2	4.72E‐3	1.13E‐2	9.83E‐3
Stomach	3.88E‐2	3.78E‐2	3.59E‐2	2.45E‐2	2.05E‐2	2.97E‐2	2.59E‐2	1.83E‐2	4.94E‐2	4.38E‐2
Brain	4.55E‐2	4.21E‐2	3.75E‐2	3.71E‐2	3.99E‐2	4.33E‐2	3.89E‐2	3.34E‐2	4.55E‐2	3.79E‐2
Gall bladder	4.49E‐2	4.22E‐2	3.74E‐2	2.85E‐2	2.41E‐2	3.38E‐2	3.09E‐2	2.07E‐2	2.98E‐2	4.43E‐2
Thyroid	4.25E‐2	4.17E‐2	3.41E‐2	2.70E‐2	1.98E‐2	–	–	1.31E‐2	–	–
Heart	3.99E‐2	3.44E‐2	2.91E‐2	2.31E‐2	1.25E‐1	8.95E‐2	1.23E‐1	5.42E‐2	3.12E‐1	9.93E‐2
Liver	2.19E‐1	1.65E‐1	1.20E‐1	8.64E‐2	3.96E‐2	4.14E‐2	4.76E‐2	2.94E‐2	6.78E‐2	5.95E‐2
Urinary bladder	1.49E‐1	2.52E‐1	1.01E0	5.44E‐2	1.10E0	8.21E‐1	3.33E‐1	2.55E‐1	1.39E0	1.16E0
Small intestine	4.12E‐2	3.92E‐2	3.19E‐2	2.76E‐2	2.85E‐2	2.93E‐2	2.52E‐2	1.97E‐2	5.28E‐2	4.51E‐2
Esophagus	4.90E‐2	4.53E‐2	3.71E‐2	2.81E‐2	2.86E‐2	3.54E‐2	2.93E‐2	2.09E‐2	4.29E‐2	4.64E‐2
Pancreas	4.46E‐2	4.29E‐2	3.74E‐2	2.75E‐2	2.17E‐2	3.26E‐2	2.76E‐2	2.00E‐2	5.29E‐2	4.81E‐2
Kidneys	4.25E‐2	4.16E‐2	3.38E‐2	2.70E‐2	1.90E‐2	3.21E‐2	2.52E‐2	1.88E‐2	4.85E‐2	4.57E‐2
Colon	4.48E‐2	4.25E‐2	3.70E‐2	2.68E‐2	2.65E‐2	4.04E‐2	2.79E‐2	2.19E‐2	4.96E‐2	5.10E‐2
Lungs	6.69E‐2	4.21E‐2	3.39E‐2	2.39E‐2	3.54E‐2	3.91E‐2	4.13E‐2	2.74E‐2	5.99E‐2	5.53E‐2
Adrenals	4.68E‐2	4.39E‐2	3.72E‐2	2.64E‐2	–	5.15E‐2	2.80E‐2	2.09E‐2	2.24E‐2	2.09E‐2
Red bone marrow	3.25E‐2	3.19E‐2	2.56E‐2	2.07E‐2	3.93E‐2	3.41E‐2	1.88E‐2	2.82E‐2	1.71E‐2	1.30E‐2
Eye lens	3.42E‐2	3.33E‐2	3.16E‐2	2.19E‐2	1.90E‐2	1.23E‐2	1.85E‐2	2.85E‐2	1.49E‐2	–

^*^Female.

^**^Male.

According to this table, the minimum amount of absorbed dose belonged to the skin for all ages and types of phantoms. The liver and urinary bladder received the highest amount of dose in UF phantoms. While in IT'IS phantoms, heart and urinary bladder had the maximum absorbed dose among the other organs. Based on the results, the amount of absorbed dose decreased with increasing the distance of organs, which was strongly related to the anatomy and type of the phantom. According to the calculations, the liver absorbed dose of UF 8‐year‐old was higher than that of IT'IS 8‐year‐old female and male models by a factor of 3.46 and 5.61, respectively; while for UF 11‐ and 14‐year‐old, the liver dose was 1.77 and 1.45 times greater than that of IT'IS 11‐ and 14‐year‐old phantoms, respectively.

## 4 DISCUSSION

In this study, organ and effective doses for weight‐based whole body pediatric PET/CT imaging were calculated. In radiation protection dosimetry, anatomical variations associated with body shape and size account for a significant portion of uncertainty in the internal and external dose estimates derived from 50th‐percentile phantoms. Although reference phantoms are valuable, they have limited use in assigning organ doses for the individual patients with a body’ shape and size far from the 50th height/weight percentile. Since the reference phantoms do not accurately reflect the range of variation in sizes of patients, the contribution of the present work is to determine the dose values for additional sizes of patients, so the practitioner could choose the model that most closely matches his/her patient in terms of height and weight. Thus, a wider range of patient sizes has been analyzed in order to improve the accuracy of dose estimates. Due to these issues, two different sets of pediatric phantoms were investigated in the whole body PET/CT imaging: UF reference and IT'IS nonreference phantoms.

As suggested in the handbook of anatomical phantoms for future needs, a comparison between phantoms is important to quantify the significance of dose uncertainty due to variations in organ topology.^36^ Efforts in developing database for radiation dose would also be used to identify patients whose cumulative lifetime ionizing radiation dose from frequent imaging has reached higher levels. This information may help determining when alternative imaging could be considered.^37^ In addition, library of pediatric dose estimates could help the manufacturer to improve the CT machine and reasonably maximizing the benefit to risk ratio. As known, the benefit to risk ratio of CT imaging should be as high as reasonably achievable. Accordingly, further work and increased scrutiny on dose issues specifically for the patient in CT practice is recommended to optimize dose values. In this regard, CT manufacturers were challenged to focus on dose issue and improve their scanners’ dose efficiency. This trend is expected to be fulfilled based on technical innovations and advances, so CT can be a valuable very low‐dose diagnostic modality.^38^

Although these considered PET/CT protocols result in lower estimated effective doses for younger patients, it does not address the children's higher radiosensitivity, so that the same effective dose results in a greater cancer risk for children than adults. As known, effective dose is the protection quantity, and its main use is to assess dose for planning and optimization in radiological protection.^29^ On the other hand, besides the magnitude of the dose, cancer risk may depend on the type of cancer, the quality of the radiation, the dose‐rate, the age, and gender of the exposed person.^17^ Therefore, effective dose does not suffice to define pediatric radiation‐induced risks.

### 4.1 Comparison of absorbed dose of ^18^F‐FDG with ICRP values

Calculated absorbed doses and effective doses of ^18^F‐FDG for UF and IT'IS phantoms were compared with those provided in ICRP publication 128 for 1‐, 5‐, 10‐, and 15‐year‐old children.^26^ This comparison is given in Table 11. For better evaluation, the closest ages of phantoms were considered and organ absorbed doses of IT'IS 5‐year‐old with ICRP 5‐year‐old, IT'IS and UF 11‐year‐old with ICRP 10‐year‐old, and IT'IS and UF 14‐year‐old with ICRP 15‐year‐old were compared together. According to Table 11, a good agreement was found between the data calculated in this study and ICRP publication 128 reference values. Our results were in the same magnitude as those of ICRP publication 128, so the calculation procedures were validated. This comparison confirmed that in general as age increases, the absorbed dose of radiotracer decreases. The difference between absorbed dose and effective dose for various phantoms declared that the phantom type has an important role in dosimetry calculations. It seems that anatomical differences cause dose uncertainty at the same ages of pediatric models. Since the amount of urinary bladder contents (i.e., empty, partially filled, or full bladder) affects its size, the maximum difference in organ absorbed doses between phantoms with the same age was related to urinary bladder wall. Disregarding urinary bladder, the maximum and minimum relative difference in absorbed dose between IT'IS and ICRP phantoms for 5‐year‐old was related to gonads (96.74%) and brain (1.09%), respectively. The relative difference in effective dose for two 5‐year‐old phantoms was 77.86%. The relative difference in absorbed dose between IT'IS and UF of 11‐year‐old phantoms varied from 2.68% (for brain) to 34.48% (for kidneys). Additionally, the maximum discrepancy in effective dose was 78.38%. For 14‐year‐old phantoms, the highest and lowest relative difference of absorbed dose was for gall bladder wall (88.18%) and liver (5%), respectively. While the uncertainty of effective dose was 10.83%.

**TABLE 11:** Comparison of ^18^F‐FDG absorbed dose (mGy/MBq.h) of UF and IT'IS phantoms with ICRP data

	5‐year‐old		11‐year‐old			14‐year‐old		
	IT'IS	ICRP	IT'IS	UF	ICRP (10 years)	IT'IS	UF	ICRP (15 years)
Gonads	8.46E‐2	4.30E‐2	2.87E‐2	3.27E‐2	2.70E‐2	1.56E‐2	2.40E‐2	1.80E‐2
Skin	1.13E‐2	2.60E‐2	1.40E‐2	8.82E‐03	1.50E‐2	4.72E‐3	6.93E‐3	9.60E‐3
Stomach	4.94E‐2	3.50E‐2	2.05E‐2	3.59E‐2	2.20E‐2	1.83E‐2	2.45E‐2	1.40E‐2
Brain	4.55E‐2	4.60E‐2	3.99E‐2	3.75E‐2	4.10E‐2	3.34E‐2	3.71E‐2	3.90E‐2
Gall bladder	2.98E‐2	2.90E‐2	2.41E‐2	3.74E‐2	1.80E‐2	2.07E‐2	2.84E‐2	1.10E‐2
Heart	3.12E‐1	2.10E‐1	1.25E‐1	1.20E‐1	1.30E‐1	5.42E‐2	8.64E‐2	8.70E‐2
Liver	6.78E‐2	6.30E‐2	3.96E‐2	4.19E‐2	4.20E‐2	2.94E‐2	2.80E‐2	2.80E‐2
Bladder	1.39E0	3.40E‐1	1.10E0	1.44E‐2	2.50E‐1	2.55E‐1	5.44E‐2	1.60E‐1
Small intestine	5.28E‐2	4.00E‐2	2.85E‐2	3.19E‐2	2.50E‐2	1.97E‐2	2.76E‐2	1.60E‐2
Esophagus	4.29E‐2	3.50E‐2	2.86E‐2	3.71E‐2	2.20E‐2	2.09E‐2	2.81E‐2	1.50E‐2
Pancreas	5.29E‐2	4.00E‐2	2.17E‐2	0.03735	2.60E‐2	2.00E‐2	2.75E‐2	1.60E‐2
Thymus	5.46E‐2	3.50E‐2	2.67E‐2	3.59E‐2	2.20E‐2	1.96E‐2	2.89E‐2	1.50E‐2
Kidneys	4.85E‐2	4.50E‐2	1.90E‐2	3.41E‐2	2.90E‐2	1.88E‐2	2.70E‐2	2.10E‐2
Colon	4.96E‐2	3.90E‐2	2.65E‐2	3.70E‐2	2.50E‐2	2.19E‐2	2.68E‐2	1.60E‐2
Lungs	5.99E‐2	6.20E‐2	3.54E‐2	3.39E‐2	4.10E‐2	2.74E‐2	2.39E‐2	2.90E‐2
Red bone marrow	3.93E‐2	3.20E‐2	1.71E‐2	2.57E‐2	2.10E‐2	1.30E‐2	2.07E‐2	1.40E‐2
Effective dose (mSv/MBq)	9.96E‐2	5.60E‐2	6.60E‐2	3.58E‐2	3.70E‐2	2.66E‐2	3.34E‐2	2.40E‐2

As Xie et al. stated, discrepancies between geometries and tissue contours might affect *S*‐values, and therefore, absorbed dose; so that the ratios of *S*‐value between stylized and voxel phantoms varied between 0.21 and 2.32. On the other hand, for two voxel models with different resolutions, there was a good agreement between the ratios of *S*‐value and given their results, voxel size did not affect dose values significantly.^21^

### 4.2 Absorbed dose and effective dose from whole body PET/CT

**FIGURE 3:**
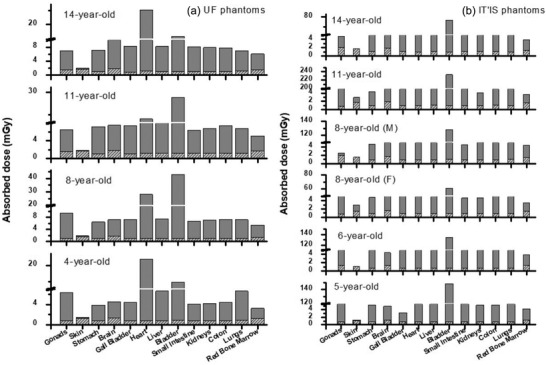
Comparison of absorbed dose due to PET and CT for (a) UF and (b) IT'IS phantoms

In this study, weight‐based scanning protocols for whole body PET/CT imaging of pediatric patients were applied, so that the injected ^18^F‐FDG activity of 6 MBq/kg^39^ and CT tube loadings of 20 mAs for 4, 5, and 6 years old, 25 mAs for 8 years old, and 30 mAs for 11 and 14 years old phantoms were considered.^32^ Therefore, the absorbed dose from CT and PET components was calculated (in mGy) based on the specifications of each phantom. The comparison between absorbed doses of PET and CT is illustrated in Figure 3 for UF and IT'IS phantoms.

According to the results, the ratio of absorbed dose from PET to CT scan was minimum for skin among the other organs. So that this ratio was 1 for UF 4‐year‐old and IT'IS 6‐year‐old and it was 1.96 for IT'IS 8‐year‐old female phantom. While the maximum value was observed for bladder and varied in the range of 17.78 for UF 4‐year‐old to 230.89 for IT'IS 11‐year‐old phantom depending on their bladder contents.

**FIGURE 4:**
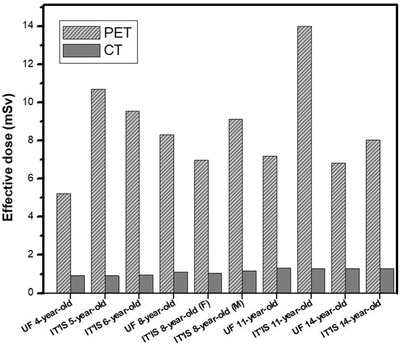
Comparison of effective dose due to PET and CT for UF and IT'IS phantoms

Effective doses of PET/CT imaging in terms of mSv for all phantoms are provided in Figure 4. Based on these data, PET contributes up to 90% of the effective dose values. It is worth mentioning that total effective dose (the sum of PET and CT component) of IT'IS 11‐year‐old phantom had the largest value of 15.28 mSv, and the UF 4‐year‐old phantom had the lowest value of 6.15 mSv. The total effective dose of IT'IS 8‐year‐old male phantom was 1.28 and 1.09 times higher than that of IT'IS 8‐year‐old female and UF 8‐year‐old phantom, respectively. Moreover, total effective doses of IT'IS 11‐ and 14‐year‐old phantoms were 1.79 and 1.15 times higher than those of UF phantoms with the same ages, respectively.

According to the outcomes, the differences in the amount of organ and effective doses of nonreference phantoms (IT'IS), using data of UF reference phantoms, are significant. This means that since anatomical data of nonreference phantoms have not been adjusted based on ICRP publication 89, they have many anatomical differences (e.g., organ location, organ size, and organ mass) with 50th percentile phantoms with the same age, which causes discrepancies in dose values. Given the fact that absorbed and effective doses vary according to patient sizes, this investigation also emphasizes on the importance of incorporating a library of phantoms with various sizes into the database in order to help to individualize dose calculations.

## 5 CONCLUSION

Monte Carlo method is a powerful tool for internal radiation dosimetry calculation, where accurate dose evaluation is grounded on reliable computational phantoms that simulate the internal anatomic geometries and physical characteristics of the human body. The results of absorbed dose and effective dose calculations showed the discrepancies related to the diversity of anatomy of the models (size, shape, and position of the organs). Some physical characteristics of organs/tissues in populations of different ages, shapes, and sizes may be substantially different and present different radiosensitivity and radiation risks. So, evaluation of age‐, shape‐, and size‐dependent absorbed dose using realistic anatomical computational phantoms is recommended.

For this purpose, our study was conducted to evaluate the radiation dose from whole body PET/CT procedures using UF and IT'IS computational phantoms and to investigate the uncertainty dosimetric data. This database of organ absorbed dose and effective dose for ^18^F‐FDG can be used for radiation risk assessment of pediatric patients in clinical routine.

## CONFLICTS OF INTEREST

The authors have no conflicts to disclose.

## AUTHOR CONTRIBUTIONS

N. Mohammadi: Project administration; Supervision; Conceptualization; Investigation; Methodology; Validation; Data curation of PET; Formal analysis; Visualization; Writing‐Original draft preparation; Writing‐Reviewing and Editing. P. Akhlaghi: Conceptualization; Investigation; Methodology; Validation; Data curation of CT; Formal analysis; Visualization; Writing ‐ review and editing.

## References

[1] Parisi MT , Bermo M , Alessio AM , Sharp SE , Gelfand MJ , Shulkin BL . Optimization of pediatric PET/CT. Semin Nucl Med. 2017;47(3):258‐274. 28417855 10.1053/j.semnuclmed.2017.01.002

[2] Beyer T , Antoch G , Muller S . Acquisition protocol considerations for combined PET/CT imaging. J Nucl Med. 2004;45(1):25S‐35S. 14736833

[3] Steinert HC , Von Schulthess GK . Initial clinical experience using a new integrated in‐line PET/CT system. Br J Radiol. 2002;75(9):S36‐S38. 12519734 10.1259/bjr.75.suppl_9.750036

[4] Wahl RL . Why nearly all PET of abdominal and pelvic cancers will be performed as PET/CT. J Nucl Med. 2004;45(1):82S‐95S. 14736839

[5] Fahey FH . Dosimetry of pediatric PET/CT. J Nucl Med. 2009;50(9):1483‐1491. 19690036 10.2967/jnumed.108.054130

[6] Uslu L , Doing J , Link M , Rosenberg J , Quon A , Daldrup‐Link HE . Value of 18F‐FDG PET and PET/CT for evaluation of pediatric malignancies. J Nucl Med. 2015;56(2):274‐286. 25572088 10.2967/jnumed.114.146290

[7] Tatsumi M , Miller KH , Wahl RL . 18F‐FDG PET/CT in evaluating non‐CNS pediatric malignancies. J Nucl Med. 2007;48(12):1923‐1931. 18056332 10.2967/jnumed.107.044628

[8] Treves ST , Baker A , Fahey FH , et al. Nuclear medicine in the first year of life. J Nucl Med. 2011;52(6):905‐925. 21622894 10.2967/jnumed.110.084202

[9] Kleis M , Daldrup‐Link H , Matthay K , et al. Diagnostic value of PET/CT for the staging and restaging of pediatric tumors. Eur J Nucl Med Mol Imaging. 2009;36(1):23‐36. 18719909 10.1007/s00259-008-0911-1

[10] Gelfand MJ , Sharp SE , Treves ST , Fahey FH , Parisi MT , Alessio AM . Estimated cumulative radiation dose from PET/CT in children with malignancies. Pediatr Radiol. 2010;40(10):1712‐1713. 20706713 10.1007/s00247-010-1794-4

[11] Sgouros G , Frey EC , Bolch WE , Wayson MB , Abadia AF , Treves ST . An approach for balancing diagnostic image quality with cancer risk: application to pediatric diagnostic imaging of 99mTc‐dimercaptosuccinic acid. J Nucl Med. 2011;52(12):1923‐1929. 22144506 10.2967/jnumed.111.092221 PMC3290866

[12] Robbins E . Radiation risks from imaging studies in children with cancer. Pediatr Blood Cancer. 2008;51(4):453‐457. 18465805 10.1002/pbc.21599

[13] Steinert M , Weiss M , Gottlöber P , et al. Delayed effects of accidental cutaneous radiation exposure: fifteen years of follow‐up after the Chernobyl accident. J Am Acad Dermatol. 2003;49(3):417‐423. 12963904 10.1067/s0190-9622(03)02088-7

[14] Xie T , Zaidi H . Evaluation of radiation dose to anthropomorphic paediatric models from positron‐emitting labelled tracers. Phys Med Biol. 2014;59(5):1165‐1187. 24557029 10.1088/0031-9155/59/5/1165

[15] Miglioretti DL , Johnson E , Williams A , et al. The use of computed tomography in pediatrics and the associated radiation exposure and estimated cancer risk. JAMA Pediatr. 2013;167(8):700‐707. 23754213 10.1001/jamapediatrics.2013.311 PMC3936795

[16] Xie T , Lee C , Bolch WE , Zaidi H . Assessment of radiation dose in nuclear cardiovascular imaging using realistic computational models. Med Phys. 2015;42(6):2955‐2966. 26127049 10.1118/1.4921364 PMC5148206

[17] Committee on the Biological Effects of Ionizing Radiation; National Research Council of the National Academies . Health Risks from Exposure to Low Levels of Ionizing Radiation. BEIR VII, Phase 2. National Academies Press; 2005. 25077203

[18] Papadimitroulas P , Erwin WD , Iliadou V , Kostou T , Loudos G , Kagadis GC . A personalized, Monte Carlo‐based method for internal dosimetric evaluation of radiopharmaceuticals in children. Med Phys. 2018;45(8):3939‐3949. 10.1002/mp.13055 29920693

[19] Quinn BM , Gao Y , Mahmood U , et al. Patient‐adapted organ absorbed dose and effective dose estimates in pediatric 18F‐FDG positron emission tomography/computed tomography studies. BMC Med Imaging. 2020;20(1):1‐9. 10.1186/s12880-020-0415-4 PMC6988339 31996149

[20] Petoussi‐Henss N , Zankl M , Hoeschen C , Nosske D . Voxel or MIRD‐type model: a sensitivity study relevant to nuclear medicine. World Congress on Medical Physics and Biomedical Engineering. 2007:2061‐2064.

[21] Xie T , Bolch WE , Lee C , Zaidi H . Pediatric radiation dosimetry for positron‐emitting radionuclides using anthropomorphic phantoms. Med Phys. 2013;40(10):1‐14. 10.1118/1.4819939 24089923

[22] Bolch W , Lee C , Wayson M , Johnson P . Hybrid computational phantoms for medical dose reconstruction. Radiat Environ Biophys. 2010;49(2):155‐168. 20039051 10.1007/s00411-009-0260-x PMC2855752

[23] Lee C , Williams JL , Bolch WE . Whole‐body voxel phantoms of paediatric patients—UF Series B. Phys Med Biol. 2006;51(18):4649‐4661. 16953048 10.1088/0031-9155/51/18/013

[24] Lee C , Williams JL , Lee C , Bolch WE . The UF Series of tomographic computational phantoms of pediatric patients. Med Phys. 2005;32(12):3537‐3548. 16475752 10.1118/1.2107067

[25] Christ A , Kainz W , Hahn EG , et al. The Virtual Family—development of surface‐based anatomical models of two adults and two children for dosimetric simulations. Phys Med Biol. 2009;55(2):23‐38. 10.1088/0031-9155/55/2/N01 20019402

[26] ICRP 128 . Radiation Dose to Patients from Radiopharmaceuticals: A Compendium of Current Information Related to Frequently Used Substances. 2015. 10.1177/0146645314558019 26069086

[27] Nuclear Data Center at KAERI. https://atom.kaeri.re.kr/nuchart/

[28] Bolch WE , Eckerman KF , Sgouros G , Thomas SR . MIRD pamphlet no. 21: a generalized schema for radiopharmaceutical dosimetry—standardization of nomenclature. J Nucl Med. 2009;50(3):477‐484. 19258258 10.2967/jnumed.108.056036

[29] Khursheed A , Hillier MC , Shrimpton PC , Wall BF . Influence of patient age on normalized effective doses calculated for CT examinations. Br J Radiol. 2002;75(898):819‐830. 12381691 10.1259/bjr.75.898.750819

[30] Rafat Motavalli L , Hoseinian Azghadi E , Miri Hakimabad H , Akhlaghi P . Pulmonary embolism in pregnant patients: assessing organ dose to pregnant phantom and its fetus during lung imaging. Med Phys. 2017;44(11):6038‐6046. 28869670 10.1002/mp.12558

[31] Ebrahimi‐Khankook A , Akhlaghi P , Vejdani‐Noghreiyan A . Studying the lung dose uncertainty during chest CT scans using phantoms with statistical lung volumes and shapes. J Radiol Prot. 2019;39(2):443‐454. 30673649 10.1088/1361-6498/ab0116

[32] Alessio AM , Kinahan PE , Manchanda V , Ghioni V , Aldape L , Parisi MT . Weight‐based, low‐dose pediatric whole‐body PET/CT protocols. J Nucl Med. 2009; 50:1570‐1577. 19793734 10.2967/jnumed.109.065912

[33] Barrington SF , Begent J , Lynch T , et al. Guidelines for the use of PET–CT in children. Nucl Med Commun. 2008;29(5):418‐424. 18391724 10.1097/MNM.0b013e3282f767b2

[34] Briesmeister JF . MCNP 4C General Monte Carlo n‐Particle Transport Code Version 4C. Report LA‐13709‐M. Los Alamos National Laboratory; 2000.

[35] ICRP103 . The 2007 Recommendations of the International Commission on Radiological Protection. 2007.

[36] Xu XG , Eckerman KF . Handbook of Anatomical Models for Radiation Dosimetry. Taylor & Francis; 2009:1‐760.

[37] Ghita M . Computer Simulations to Estimate Organ Doses from Clinically Validated Cardiac, Neuro, and Pediatric Protocol for Multiple Detectors Computed Tomography Scanners. PhD thesis. University of Florida; 2009.

[38] Kalender WA. Dose in X‐ray computed tomography. Phys Med Biol. 2014;59:R129‐150. 24434792 10.1088/0031-9155/59/3/R129

[39] Stauss J , Franzius C , Pfluger T , et al. Guidelines for 18F‐FDG PET and PET‐CT imaging in paediatric oncology. Eur J Nucl Med Mol Imaging. 2008; 35:1581‐1588. 18536914 10.1007/s00259-008-0826-x

